# 

**Published:** 1862-07

**Authors:** 


### CHICAGO MEDICAL JOURNAL.

**Terms, $2.00 per Annum.**

### CONTENTS OF THE JULY NUMBER.

PAGE.

ORIGINAL COMMUNICATIONS.

Hernia Cerebri. By R. L. Rea, M. D., Prof. Anatomy Rush Med. College, 375

Case of Hydrops Amnii. By A. E. Johnson, M. D., of St. Anthony, Minn. 394

SELECTED.

On the Climate of the Mountain Ranges of California in relation to the Treatment of Phthisis. By James Blake, M. D.................. 396

Considerations in reference to Healthy in Contra-Distinction to Diseased Joints. From a paper by John Swineburne, M. D., Albany, N. Y.. 400

Small Pox and Vaccination. By H. Gibbons, M. D.................... 402

Cases of Reflex and Infantile Paralysis................................ 404

EDITORIAL AND MISCELLANEOUS........................................ 406

